# Age-specific associations between intergenerational support from children and depression in middle-aged and elderly Chinese: results from the China health and retirement longitudinal study

**DOI:** 10.3389/fpsyg.2025.1621444

**Published:** 2025-07-17

**Authors:** Yongbo Lu, Jingya Zhang, Zongyang Zhou, Rongxin He, Bin Zhu, Ying Mao

**Affiliations:** ^1^School of Public Policy and Administration, Xi’an Jiaotong University, Xi'an, China; ^2^School of Health Management, Southern Medical University, Guangzhou, China; ^3^School of Public Health and Emergency Management, Southern University of Science and Technology, Shenzhen, China; ^4^SUSTech Homeostatic Medicine Institute, School of Medicine, Southern University of Science and Technology, Shenzhen, China

**Keywords:** intergenerational support, children, depression, middle-aged and elderly, Chinese

## Abstract

**Objectives:**

To assess the age-specific associations between intergenerational support from children and depression in middle-aged and elderly Chinese.

**Methods:**

In total, 11,398 adults aged ≥45 who participated in the China Health and Retirement Longitudinal Study (CHARLS) 2018 were included. Depression was assessed using the Center for Epidemiological Studies Depression Scale (CES-D). Logistic regression analysis was performed to examine the effects of offline companionship, online companionship, money support, and goods support from children on the depression status of middle-aged and elderly people.

**Results:**

Among people aged 80 and above, offline companionship is associated with a reduced risk of depression (OR = 0.573, 95% CI: 0.372, 0.883). However, online companionship may be associated with an increased risk of depression in individuals aged 45–60. Money support was found to have a positive association with depression in individuals aged 45–60 (OR = 1.182, 95% CI: 1.005, 1.389), but a negative association in those aged 60–80 (OR = 0.767, 95% CI: 0.632, 0.930). Goods support is associated with a decreased risk of depression in individuals aged 45–80.

**Conclusion:**

For individuals aged 45–60, children can offer valuable goods support to mitigate parental depression. In the 60–80 age group, both money and goods support are essential. For those over 80, increased offline companionship is recommended.

## 1 Introduction

In recent decades, the global elderly population has experienced substantial growth (Fong et al., 2021) China’s population aging process accelerated in the late 1970s and has continued at an annual rate of about 3.2% since then. While this process took more than 45 years in developed countries, it occurred in China in about 27 years, and this trend is likely to persist for an extended period (Bao et al., 2022). According to the latest Seventh National Population Census of China (Tu et al., 2022), as of 2020, the population aged 60 and over reached 264.02 million, accounting for 18.70% of the total population. China, with one fifth of the world’s elderly population (Chen et al., 2022), has become a typical aging country in the world. According to United Nations criteria, the older population in China is projected to surpass 300 million by 2025, signifying a transition from a mildly to a moderately aging status (Hu et al., 2021).

China’s aging population has brought complex social challenges (Man et al., 2021), including mental health concerns that place pressure on both families and society. Specifically, the incidence of major depressive disorder rises with age (Kok and Reynolds, 2017). According to the Blue Book of Aging Development in China 2024 (Foundation, China Development Research, 2024), 26.4% of Chinese older adults present with varying levels of depressive symptoms, among whom 6.2% report moderate to severe manifestations. Research discovered that approximately one-fifth of the 950 participants aged over 60 from 22 locations in China exhibited symptoms of depression (Chen et al., 2021). There is also a physiological scientific rationale for the elevated prevalence of depression among the elderly. Aging-related processes impact the integrity of the frontal striatal pathway, amygdala, and hippocampus, potentially increasing susceptibility to depression (Alexopoulos, 2005). Late-life depression may also give rise to additional complications, including cognitive decline, which can result in reduced quality of life and even an elevated risk of mortality among older adults (Gutzmann and Qazi, 2015).

These mental health issues in the elderly must be taken seriously because of their significant impact on both the individual and society. At the individual level, mental health tends to act on a range of socially engaged behaviors (Parra-Rizo et al., 2022), such as daily living activities, interpersonal network communication, sports and physical activity, further impacting the quality of life (Dpto and Parra-Rizo., 2020; Hussenoeder et al., 2021) of older persons. This should not be overlooked, as quality of life has a direct impact on well-being. On a societal scale, the mental health of older persons poses a higher challenge to the health economy and institutional security. Research (de Oliveira et al., 2019) data suggests that the average annual incremental cost of healthcare spending for older adults with depression and chronic conditions is $7,940 compared to older adults without these conditions. Worse, it can even trigger suicidal ideation in the elderly. Not only is it a heavy socio-economic burden, but it also places higher demands on the national health protection system for the elderly, a segment of the population that has to need more social support. Given the severity of late-life depression and its multifaceted consequences, identifying effective protective factors and intervention strategies has become a critical public health priority, particularly in rapidly aging societies like China.

In the context of China’s familial culture and limited formal care infrastructure, intergenerational support from adult children emerges as a pivotal factor against depression in older adults, even more important than social support (Chen et al., 2011; Silverstein et al., 2006). The Family Communication Patterns Theory (Koerner and Fitzpatrick, 2006) posits that intergenerational support impacts the health behavior and outcomes of the elderly. This support operates through multiple mechanisms: it enhances social integration, provides a sense of purpose and value, buffers against life stressors, and facilitates access to healthcare resources (Guo et al., 2009; Silverstein et al., 2006). The importance of intergenerational support is particularly pronounced in China. First, the traditional concept of ‘xiao’ (filial piety) creates strong normative expectations for adult children to care for aging parents, making such support a primary source of elderly well-being (Zhan and Montgomery, 2003). Second, China’s social security system remains underdeveloped relative to its rapid population aging, leaving many elderly individuals heavily dependent on family support (Chou, 2011). Empirical evidence focused on the mental health of the elderly consistently demonstrates the protective effects of intergenerational support against depression among middle-aged and elderly individuals (Almeida et al., 2009; Zheng et al., 2022; Bai et al., 2025). A cross-sectional study in China explored the dose–response relationship between intergenerational support and geriatric depression, providing additional evidence for this finding (Xie et al., 2020).

Intergenerational support (Huang and Fu, 2021) encompasses assistance from children, siblings, and parents, among others. Intergenerational support from adult children typically falls into three categories, emotional support, instrumental support, and financial support (Chen and Silverstein, 2000). Emotional support often takes the form of traditional offline companionship, involving face-to-face interaction, which plays a fundamental role in combating the social isolation and loneliness that often precipitate late-life depression (Cacioppo et al., 2010). These interactions provide elderly parents with a sense of being valued and loved, directly countering feelings of worthlessness—a core symptom of depression (Park et al., 2014). Given that many adult children work away from home, online companionship through platforms like WeChat and short message service has emerged as a supplementary method to offer continuous emotional connection, facilitated by the widespread use of the Internet (Fang et al., 2020). Regular online communication specifically reduces rumination and negative thought patterns by providing elderly parents with consistent emotional anchoring and cognitive stimulation. Instrumental support involves adult children offering caregiving and nursing assistance to older individuals when they require help with daily tasks (Liu et al., 2022). By assisting with daily activities and healthcare navigation, adult children help elderly parents maintain functional independence, which is strongly linked to self-esteem and mental health (Krause, 2007). This support is particularly crucial in preventing the cascade from physical limitations to learned helplessness and subsequent depression (Bruce, 2001). Financial support can encompass both monetary contributions and tangible goods, which functions as both a material and symbolic resource in preventing depression. Materially, it alleviates economic stress and enables access to healthcare, nutrition, and social activities—all protective factors against depression (Blazer, 2003). Symbolically, financial support from children represents the continuation of traditional filial obligations, providing elderly parents with a sense of security and family cohesion (Silverstein et al., 2006).

Some studies (Cornect-Benoit et al., 2020; Gualano et al., 2018) have demonstrated that the existence of intergenerational support from adult children can enhance healthy brain aging in the elderly. These forms of support(Patterson, 2022), can effectively enhance the health status of elderly individuals (Li and Guo, 2022), including their mental well-being (Wu et al., 2018). Again, considering the three traditional categories of child support, we consider the real situation of Chinese society and refine the classification of traditional child support, forming a theoretical framework of the role of offline companionship (under instrumental support and emotional support), online companionship (under emotional support), money support (under financial support), and goods support (under financial support) on the mental health of middle-aged and elderly people. However, as elderly individuals of varying ages undergo changes in physical functioning, social engagement, and more, their mental states may also vary. Consequently, different types of support from adult children may yield differing effects on enhancing their mental health status. Recent global reviews (Whear et al., 2023) have also suggested that different child support may have age-specific effects on the elderly, in view of the different roles they play in the family and society at different stages of life. However, there is little evidence evaluating the differential impact of specific types of support at different ages in China. Given the groundwork that has been laid by previous studies exist in other countries or contexts, in the context of China’s cultural emphasis on filial piety, it is imperative to comprehensively assess the most efficacious forms of support for alleviating depression in older individuals across diverse age groups.

Therefore, we aim to assess the age-specific associations between intergenerational support from adult children and depression in middle-aged and elderly individuals in China. We seek to investigate which forms of intergenerational support from adult children are most advantageous for the mental well-being of older adults at varying ages. To accomplish this, we focus on the following questions:

Q1: Can offline companionship, as traditional emotional support, and instrumental support, affect depression status of middle-aged and elderly people?

Q2: Can online companionship, which has become prevalent with the growth of the Internet, impact the depressive status of middle-aged and elderly adults?

Q3: Can money support influence the depression experienced by middle-aged and elderly individuals?

Q4: Can goods support influence the depressive symptoms of middle-aged and elderly individuals?

## 2 Materials and methods

### 2.1 Data source and sample

The data in this study were obtained from the China Health and Retirement Longitudinal Survey (CHARLS) 2018. The sampling process involved stratification by region (Eastern/Central/Western), urban/rural status, and GDP per capita, with 150 county-level units selected via PPS from mainland China (excluding Tibet). Within each county, 3 primary sampling units were selected, followed by household selection using CHARLS-GIS mapping software, and finally randomly selecting one person aged 45 + per household plus their spouse. By Wave 4 (CHARLS, 2018), the sample had expanded to 19,817 individuals through the inclusion of refreshment samples (those aged 40–44 at baseline who reached eligibility) and newly contacted age-eligible respondents. This data is used to analyze China’s aging population and to foster interdisciplinary research on aging. The survey encompassed fundamental demographic data about the participants and their families, interfamily transfer payments, the health status of the participants, healthcare and insurance, employment, income, expenses, assets, and more. The survey employed a stratified multi-stage probability proportional to size random sampling methodology. For a more comprehensive description of the study’s design and sampling procedure, please refer to the CHARLS cohort profile (Zhao et al., 2012). Citations for the data used in the study can be accessed in these official websites: https://charls.charlsdata.com/pages/Data/2018-charls-wave4/zh-cn.html.

**Figure 1:**
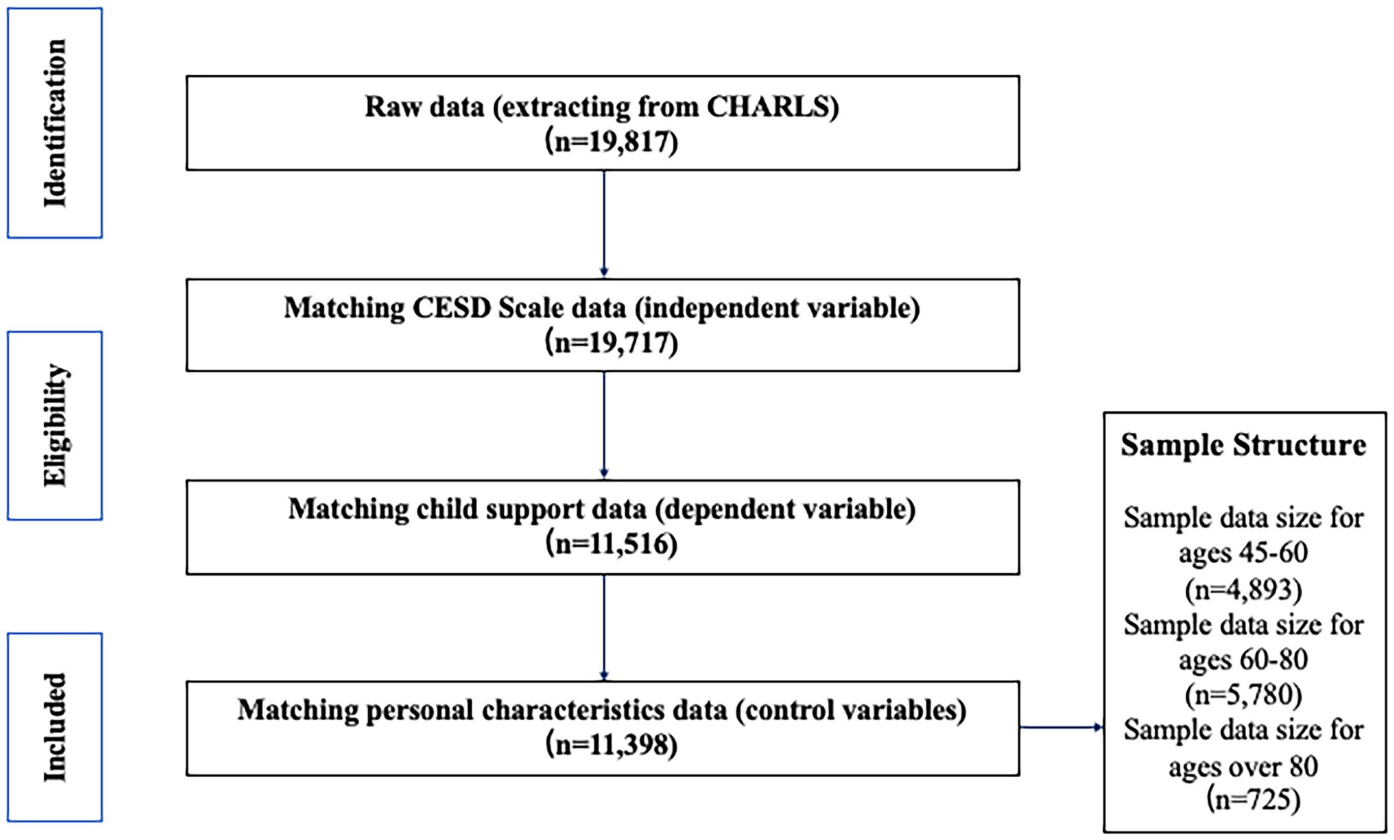
Sample selection process. CESD, Center for Epidemiologic Studies Depression Scale.

We determined the actual age of older individuals by using the question in the questionnaire, “What is your actual date of birth?” along with the survey year (2018). The actual age was calculated as the difference between the survey year and the respondent’s year of birth. On the basis of CHARLS 2018 raw data 19,817, we excluded records with missing values. Excluding cases with missing CESD scale (dependent variable) measures, 19,717 records were retained; excluding cases with missing intergenerational support (independent variable) scale measures, 11,516 records were retained; excluding cases with missing personal characteristics (control variable) measures, 11,398 records were retained. The process of elimination is shown in Figure 1. Missing values and responses such as “do not know” or “not applicable” were excluded from the sample.

### 2.2 Measurements

#### 2.2.1 Depression

Depressive symptoms were assessed using the 10-item Center for Epidemiological Studies Depression (CES-D) scale. Studies have confirmed that the CESD scale has reliable reliability and validity among middle-aged and older people in China. There was a total of 10 items, and after excluding the records of missing values, scores were assigned positively to 8 items and negatively to 2 items, and the values assigned to the 10 items were summed to the CESD scale score (see Supplementary file 1 for details of the assignments). In the CHARLS 2018 survey, CESD’s Cronbach’s alpha = 0.784 proved to have good internal consistency. The scores for all items were summed, resulting in a total score ranging from 0 to 30. The CES-D-10 comprises 10 questions about the participant’s experiences during the past week. These questions assess feelings of irritability, inattention, depression, and hopefulness about the future. A score of ≥10 indicates the presence of depression, while the opposite score signifies no depression (1 = Depression, 0 = Normal). Furthermore, In the CHARLS 2018 survey, CESD’s Cronbach’s alpha = 0.784 proved to have good internal consistency.

#### 2.2.2 Intergenerational support from children

CHARLS contained a variety of information related to intergenerational support from adult children, including offline companionship, online companionship, money support and goods support. In cases where families had multiple children, we aggregated the support given by all children. These support variables gauge the cumulative support received by middle-aged and elderly individuals from all their children, rather than evaluating support on an individual child basis.

Offline companionship assessed the amount of time individuals spent living with their children in the past year, categorized as follows: 1 = none, 2 = 1–3 months, 3 = 4–6 months, 4 = 7–9 months, 5 = 10–12 months. Online companionship inquired about the frequency of online communication via phone, text, WeChat, letter, or email when individuals were not residing with their children. This was rated on a scale of 1 = basically no, 2 = rarely, 3 = often. Money Support assessed the total monetary support received from children in the past year when individuals were not cohabiting with them. This was rated on a scale of 1 = none, 2 = 1–6,000 RMB, 3 = over 6,000 RMB. Goods Support inquired about the total value of objects received from children in the past year when individuals were not residing with them. This was rated on a scale of 1 = none, 2 = 1–6,000 RMB, 3 = over 6,000 RMB.

#### 2.2.3 Control covariates

In accordance with prior knowledge, we incorporated sociodemographic characteristics and health-related factors into our study (see Supplementary file 2 for details). Firstly, we incorporated demographic variables that have been demonstrated to impact depression and social participation. These variables included gender (1 = male, 2 = female), age (1 = 45–60 years, 2 = 60–80 years, 3 = over 80 years), place of residence (1 = the center of city/town, 2 = combination zone between urban and rural areas, 3 = village, 4 = special area), education level (1 = illiterate, 2 = primary School, 3 = junior high school, 4 = high school or specialist, 5 = bachelor’s degree or above), and marital status (1 = married and cohabiting, 2 = married but not currently cohabiting for specific reasons, 3 = divorced or widowed, 4 = never married). Secondly, we incorporated socioeconomic factors into our analysis. Social activity (Kennedy, 2019) assessed whether participants engaged in any social activities in the past month (1 = Yes, 2 = No), such as socializing with friends, participating in club activities, attending training courses, or using the Internet, among others. Additionally, we included a variable related to pensions (Pan et al., 2021), inquiring whether participants were currently receiving or expected to receive pensions in the future (1 = Yes, 2 = No). Third, we controlled for health status, as it constitutes a fundamental aspect of mental health (Shao et al., 2022). Self-rated health was assessed by asking respondents to rate their health status on a 5-point Likert scale: 1 = very good, 2 = good, 3 = fair, 4 = poor, and 5 = very poor.

### 2.3 Data analysis

At first, we initially described the characteristics of the data. The frequency, percentage and prevalence of categorical variables were reported. To assess the relationship between intergenerational support from adult children and depression in middle-aged and elderly individuals, we employed a logistic regression analysis model. We used weights for adjusting the sample data to ensure that it is representative of the nation. Odds ratios (ORs) and 95% CIs were reported for the logistic model.

Referring to the Chinese Dictionary of Population Science, we conducted subgroup analyses of age according to 45–60 years old, 60–80 years old, and 80 years old and above, representing three groups of middle-aged, lower-aged, and higher-aged seniors, respectively. Two main reasons were considered for the choice of the lower age limit for the study: firstly, the World Health Organization defines middle-aged and elderly people as those aged 45 years and above. Considering also the Chinese scenario, where the legal age of marriage is 20 (for females)/22 (for males), and the graduation age of college students is generally 22 to 23, it is likely that those over 45 years of age will begin to receive support from their children, while those under 45 years of age are still more likely to give intergenerational support to their children. Therefore, middle-aged and older adults over 45 years of age were selected for this study. We conducted subgroup analyses to assess differences among the three age groups: 45–60, 60–80, and 80 + years. On this basis, we also selected child support variables with significant effects in each age subgroup for interaction analysis. For all results, a significance level of *p* < 0.05 was used to determine statistically significant differences. Also, to ensure the robustness of all model results, we performed robustness checks, see Supplementary file 3. We altered the testing model and employed a probit regression model to further investigate the relationships between intergenerational support from adult children and depression in middle-aged and elderly Chinese individuals. All regression analyses were conducted in STATA MP 16.0 (Stata Corp LLC, Texas, US). This study followed the STROBE statement, a reporting guideline for observational research analyses.

## 3 Results

### 3.1 Participant characteristics

Table 1 displays the fundamental characteristics of the survey participants. The valid survey sample comprises 5,385 (47.25%) males and 6,085 (57.25%) females. The prevalence of depression is 26.69 and 39.90%, respectively. The age range spans from 45 to 118 years, with 4,893 (42.93%) individuals aged 45–60, 5,780 (50.71%) aged 60–80, and 725 (6.36%) over 80 years old. The prevalence rates for these three age groups are 33.80, 35.0, and 21.10%, respectively. The majority (72.14%) of respondents reside in rural areas. Over half (65.96%) have education levels of primary school or lower, while less than 1% hold a bachelor’s degree or higher. 7,754 (68.3%) are married and cohabiting with their spouses. Respondents rated their health status as follows: very good (11.34%), good (12.60%), fair (49.71%), poor (20.37%), and very poor (5.98%). Approximately half (46.20%) of the middle-aged and elderly individuals engaged in social activities in the recent past. It is noteworthy that 9,118 (80%) of the surveyed middle-aged and elderly individuals do not possess pension insurance.

**Table 1:** Descriptive statistics of variables (*N* = 11,398).

Variables	n	%	Depression number	Prevalence (%)
Offline companionship				
None	4,227	37.09	1,475	34.89
1–3 months	1,301	11.41	465	35.74
4–6 months	722	6.33	239	33.10
7–9 months	459	4.03	150	32.68
9–12 months	4,689	41.14	1,507	32.14
Online companionship				
Basically no	2,969	26.05	817	27.52
Rare	3,185	53.99	1,229	40.78
Often	5,244	46.01	1790	34.13
Money support				
None	5,382	47.22	1,664	30.92
1–6,000 RMB	4,346	38.13	1,641	37.76
over 6,000 RMB	1,670	14.65	531	31.80
Goods support				
None	4,096	35.94	1,318	32.18
1–6,000 RMB	6,722	58.98	2,394	35.61
over 6,000 RMB	580	5.09	124	21.38
Sex				
Male	5,385	47.25	1,437	26.69
Female	6,013	52.75	2,399	39.90
Age				
45–60 years	4,893	42.93	1,654	33.80
60–80 years	5,780	50.71	2029	35.10
over 80 years	725	6.36	153	21.10
Residence				
City	2,252	19.76	557	24.73
Urban–rural integration zone	875	7.68	238	27.20
Rural	8,222	72.14	3,029	36.84
Special zone	49	0.43	12	24.49
Education				
Illiterate	2,569	22.54	1,014	39.47
Primary school	4,949	43.42	1853	37.44
Junior high school	2,395	21.01	660	27.56
High school or specialist	1,376	12.07	296	21.51
Bachelor or above	109	0.96	13	11.93
Marital status				
Married (living with spouse)	7,754	68.03	2,538	32.73
Married (living apart from spouse)	806	7.07	320	39.70
Divorced or widowed	2,721	23.87	939	34.51
Never married	117	1.03	39	33.33
Health status				
Very good	1,292	11.34	198	15.33
Good	1,436	12.60	250	17.41
Fair	5,666	49.71	1,628	28.73
Poor	2,322	20.37	1,298	55.90
Very poor	682	5.98	462	67.74
Social activity				
No	6,312	55.38	1913	30.31
Yes	5,266	46.20	1923	36.52
Pension insurance				
Yes	2,280	20.00	487	21.36
No	9,118	80.00	3,349	36.73

Concerning support from children, 4,227 (37.09%) of children did not reside with their parents, while 5,244 (46.01%) lived with their parents for over 9 months. 2,969 (27.52%) of children had minimal online communication with their parents, whereas 5,244 (46.01%) frequently communicated with their parents online. Nearly half of them did not offer financial support to their parents, 4,346 (38.13%) provided financial support to their parents in the range of 1–6,000 RMB, and very few children provided financial support to their parents of 6,000 RMB or more. Over half of the children provided their parents with goods support in the range of 1–6,000 RMB, 4096 (35.94%) did not provide their parents with goods support, and very few children provided their parents with goods support of more than 6,000 RMB.

### 3.2 The relationship between intergenerational support from children and depression status in middle-aged and elderly people

Table 2 presents the results of multi-variable regression models using logistic regression. Regarding offline companionship, seniors residing with their children for over 9 months per year were less likely to experience depression (OR = 0.886, 95% CI: 10.801, 0.980), while companionship for less than 9 months showed no significant effect on depression in middle-aged and elderly individuals. Individuals with more frequent online contact with their children were more likely to experience depression (OR = 1.292, 95% CI: 1.135, 1.472 (rare); OR = 1.180, 95% CI: 1.052, 1.325 (often)). Money support from children did not significantly impact the depression of the elderly. Conversely, elderly individuals were less likely to experience depression when receiving more than 6,000 RMB per year in goods support (OR = 0.622, 95% CI: 0.492, 0.786). In the logistic regression model, women who were older, more educated, divorced, or widowed, in poorer health, less socially active, and without pension insurance were more likely to experience depression. The results of robustness tests (refer to Supplementary file 3) indicated that the significance and direction of the coefficients for dependent variables were consistent, suggesting that the estimates and results of the associations between dependent and independent variables in this study were robust and reliable.

**Table 2:** The relationship between intergenerational support from children and depression in middle-aged and elderly people.

Variables	Odds ratio	Std. err.	z	P > z	[95% Confidence interval]	
Offline companionship (ref: none)						
1–3 months	1.015	0.074	0.200	0.839	0.879	1.172
4–6 months	0.942	0.089	−0.640	0.525	0.783	1.133
7–9 months	0.864	0.099	−1.280	0.202	0.690	1.082
9–12 months	0.886	0.046	−2.360	0.018	0.801	0.980
Online companionship (ref: basically no)						
Rare	1.292	0.086	3.870	0.000	1.135	1.472
Often	1.180	0.070	2.810	0.005	1.052	1.325
Money support (ref: none)						
0–6,000 RMB	1.104	0.058	1.910	0.057	0.997	1.223
Over 6,000 RMB	0.932	0.066	−1.000	0.316	0.811	1.070
Goods support (ref: none)						
0–6,000 RMB	0.998	0.051	−0.030	0.975	0.903	1.103
Over 6,000 RMB	0.622	0.074	−3.970	0.000	0.492	0.786
Sex (ref: male)						
Female	1.752	0.083	11.880	0.000	1.597	1.922
Age (ref: 45–60 years)						
60–80 years	0.820	0.043	−3.820	0.000	0.741	0.908
Over 80 years	0.397	0.045	−8.100	0.000	0.318	0.497
Residence (ref: city)						
Urban–rural integration zone	0.985	0.096	−0.160	0.875	0.813	1.193
Rural	1.149	0.078	2.050	0.040	1.006	1.311
Special zone	0.815	0.289	−0.580	0.564	0.406	1.633
Education (ref: illiterate)						
Primary school	1.086	0.063	1.410	0.158	0.969	1.217
Junior high school	0.842	0.063	−2.300	0.021	0.728	0.975
High school or specialist	0.772	0.074	−2.700	0.007	0.640	0.932
Bachelor’s degree or above	0.579	0.183	−1.730	0.083	0.312	1.074
Marital status (ref: married (living with spouse))						
Married (living apart from spouse)	1.139	0.095	1.550	0.120	0.967	1.341
Divorced or widowed	0.935	0.053	−1.180	0.236	0.837	1.045
Never married	1.122	0.247	0.520	0.600	0.729	1.726
Health status (ref: very good)						
Good	1.195	0.126	1.690	0.092	0.971	1.470
Fair	2.193	0.185	9.330	0.000	1.860	2.587
Poor	6.450	0.581	20.690	0.000	5.406	7.695
very poor	10.806	1.247	20.630	0.000	8.619	13.547
Social activity (ref: yes)						
No	1.074	0.048	1.600	0.109	0.984	1.172
Pension insurance (ref: yes)						
No	1.272	0.092	3.320	0.001	1.103	1.466
Observation	11,398					
Pseudo R^2^	0.1203					

### 3.3 The relationship between intergenerational support from children and depression status by age groups

Table 3 presents the results of multi-variable regression models using logistic regression, stratified by age. Offline companionship from children was associated with a reduced likelihood of depression in individuals over 80 years old (OR = 0.573, 95% CI: 0.372, 0.883). Conversely, the online interaction with children increased the likelihood of depression in individuals aged 45–60 (OR = 1.702, 95% CI: 1.351, 2.143 (rare); OR = 1.339, 95% CI: 1.126, 1.592 (often)). Money support had a potential impact on individuals aged 45–80, but this effect was not significant for those aged 80 and older. Individuals aged 45–60 were more likely to experience depression when receiving money support (OR = 1.182, 95% CI: 1.005, 1.389), whereas those aged 60–80 were less likely to be depressed (OR = 0.767, 95% CI: 0.632, 0.930). Individuals aged 45–80 had a lower likelihood of depression when receiving goods support of more than 6,000 RMB (OR = 0.662, 95% CI: 0.448, 0.977 (45–60 years); OR = 0.604, 95% CI: 0.440, 0.829 (60–80 years)). The results of robustness tests (refer to Supplementary file 3) indicated that the significance and direction of the coefficients for dependent variables were consistent, suggesting that the estimates and results of the associations between dependent and independent variables in this study were robust and reliable.

**Table 3:** The relationship between children support and depression levels in middle-aged and elderly people by age groups.

Variables	Modle1 45–60 years			Modle2 60–80 years			Modle3 Over 80 years		
	*N*	OR	95% CI	*N*	OR	95% CI	*N*	OR	95% CI
Offline companionship (ref: none)	1,340			2,558			299		
1–3 months	667	1.040	0.839,1.290	591	1.036	0.844,1.271	43	0.791	0.352,1.779
4–6 months	457	0.842	0.655,1.083	242	1.131	0.842,1.52	23	1.160	0.434,3.100
7–9 months	249	0.905	0.662,1.236	191	0.838	0.594,1.182	19	0.896	0.274,2.931
9–12 months	2,180	0.977	0.828,1.154	2,168	0.907	0.791,1.039	341	0.573**	0.372,0.883
Online companionship (ref: basically no)	1,435			1,348			186		
Rare	678	1.702***	1.351,2.143	2,112	1.181	0.993,1.404	395	1.006	0.633,1.599
Often	2,780	1.339***	1.126,1.592	2,320	1.130	0.955,1.336	144	0.699	0.384,1.274
Money support (ref: none)	2,966			2,171			245		
0–6,000 RMB	1,299	1.182**	1.005,1.389	2,673	1.028	0.894,1.182	374	0.984	0.618,1.566
Over 6,000 RMB	628	1.088	0.878,1.348	936	0.767***	0.632,0.930	106	1.100	0.593,2.038
Goods support (ref: none)	2,292			1,642			162		
0–6,000 RMB	2,410	0.969	0.835,1.125	3,810	1.002	0.869,1.156	502	1.047	0.642,1.707
Over 6,000 RMB	191	0.662**	0.448,0.977	328	0.604***	0.440,0.829	61	0.439	0.171,1.129
CV		Yes			Yes			Yes	
Observation		4,893			5,780			725	
Pseudo R^2^		0.1303			0.1211			0.1059	

CV, control variables; CI, Confidence interval. *p < 0.1, **p < 0.05, ****p < 0.01.

Table 4 shows the multivariable interaction regression results of the binary logistic regression models by age. In the 45–60 age group, online companionship, money support, and goods support, as the three types of support with significant univariate effects, had insignificant interactions. Similarly, the interaction of money support and goods support, two types of support with significant univariate effects, was not significant in the 60–80 age group. For those over 80 years of age, we do not need to analyze the interaction effect since only the effect of offline companionship is significant. The results of robustness tests (refer to Supplementary file 3) indicated that the significance and direction of the coefficients for dependent variables were consistent, suggesting that the estimates and results of the associations between dependent and independent variables in this study were robust and reliable.

**Table 4:** The interaction effect of child support and depression levels in middle-aged and older adults by age groups.

Variables	45–60 years		60–80 years	
	OR	95% CI	OR	95% CI
Money support*goods support	−0.212	−0.375, 0.798	−0.074	−0.225, 0.077
Online companionship*money support	0.112	−0.295, 0.520	/	/
Online companionship* goods support	0.165	−0.192, 0.522	/	/
Online companionship*money support* goods support	−0.099	−0.320, 0.121	/	/

This table shows only the interaction effect of child support related variables. CI, Confidence interval. *p < 0.1, **p < 0.05, ****p < 0.01.

## 4 Discussion

### 4.1 Offline companionship

Middle-aged and elderly individuals who reside with their children are less susceptible to depression compared to those who do not, consistent with prior research (Brugiavini et al., 2022; Wang et al., 2021). Our research additionally affirms that the impact on depression in individuals over the age of 80 is more significant when this companionship extends beyond 9 months. Notably, for individuals over the age of 80, only cohabiting with their children showed a positive impact on alleviating depression, whereas online companionship, financial assistance, and material support exhibited no notable effect. In countries like China, where family bonds are robust, informal care provided by children serves as a valuable complement and supplement to formal care, offering older individuals vital support (Cai et al., 2024). The demand for live-in care is particularly pronounced among older adults aged 80 and above, who may encounter a range of physical function-related challenges (Cruz-Jimenez, 2017). This nearly year-round offline companionship support enables children to offer extensive informal care and attention, effectively diminishing the occurrence of depression. Additionally, cohabitating with children implies that elderly individuals may also share their living space with grandchildren. Some studies have demonstrated that grandparenting has a notable effect on depression among older individuals, and offering care to grandchildren substantially decreases depression in the elderly population (Tang et al., 2022). In conclusion, it is plausible to assert that children offer physical care services to the elderly, while grandchildren contribute to a sense of spiritual fulfillment, collectively alleviating depression among the elderly.

Given the pronounced benefits of physical companionship for individuals over 80, targeted policy interventions should prioritize facilitating in-person companionship programs for this vulnerable population. These programs could include community-based intergenerational housing initiatives that encourage multigenerational cohabitation, subsidized home care services that enable adult children to provide live-in support, and neighborhood companion programs that connect isolated elderly with trained volunteers for regular face-to-face interactions (Fakoya et al., 2020). Additionally, urban planning policies should consider creating elderly-friendly residential complexes that accommodate extended families, while rural areas could benefit from transportation subsidies enabling children to visit elderly parents more frequently (Sixsmith and Sixsmith, 2008).

### 4.2 Online communication

Online companionship with children exacerbates depression in the elderly, particularly those aged 45–60. Interestingly, in certain developed Western nations, children’s online companionship diminishes depression in middle-aged and elderly parents (Tosi and Grundy, 2018). First, from a family life cycle perspective, frequent online interactions between 45 and 60 group parents and their adult children may paradoxically signal problematic family transitions rather than positive connections. In China, children of this age group (typically aged 20–35) are just beginning to establish independence from their families of origin. Unlike elderly parents aged 60+, whose children have typically achieved stable independence, the 45–60 cohort’s children are navigating critical life transitions—career establishment, marriage, home purchase, and childrearing—often with significant difficulties (Hu and Scott, 2016; Xiao and Cooke, 2012). The high frequency of online contact may therefore reflect parental anxiety about their children’s struggles rather than meaningful emotional support. This interpretation is supported by research on ‘reverse intergenerational support’ in China, where middle-aged parents continue providing substantial assistance to adult children well into their 30s due to housing costs, childcare needs, and employment instability (Song et al., 2012).

This anxiety is amplified by China’s rapid urbanization, which has created a dual burden for families. Geographically, labor migration has separated millions of families, with adult children relocating to first-tier cities for employment opportunities while parents remain in smaller cities or rural areas (Chen and Liu, 2009). More critically, these migrant children face intense survival pressures in urban centers. The online interactions may thus serve as conduits for worry and stress transmission rather than comfort. In contrast, Western contexts characterized by individualistic values and earlier independence norms enable parents to psychologically adapt to children’s departure from the parental home, allowing them to derive genuine emotional satisfaction from voluntary online contact (Fingerman et al., 2020; Tosi and Albertini, 2019). The cultural expectation of interdependence in China transforms what could be supportive communication into anxiety-inducing monitoring of children’s unresolved challenges.

On the other hand, research (Lu et al., 2022) indicates that elderly individuals in low- and middle-income countries are more prone to experiencing digital exclusion compared to their high-income counterparts. Digital exclusion among the elderly results from various obstacles, including reluctance to adopt the Internet, financial constraints preventing Internet access, and insufficient digital literacy and skills. The potential factors contributing to the contrasting findings in China are the generally low literacy levels (Zhu and Ye, 2020) of the current middle-aged and elderly generation and their limited proficiency in using electronic devices. Many of China’s elderly individuals faced significant challenges in accessing the Internet (McCloud et al., 2016) owing to diminished learning capabilities (Kurdziel et al., 2017). Excessive online communication widens the psychological divide and fosters a sense of falling behind, ultimately heightening the risk of depression (Wang and Geng, 2019). The majority of children who engage in extended online interactions with the elderly are typically employed away from home. This physical separation already leads to frustration among the elderly, and the resulting detachment from online communication further compounds the psychological distance, consequently elevating the risk of depression in this age group. In conclusion, it is plausible to assert that this current generation of elderly individuals has not yet effectively adapted to engaging with family members via the Internet.

These findings highlight critical intervention needs. While China has initiated digital inclusion programs such as the ‘Internet Plus’ strategy for elderly services, current efforts remain insufficient. Effective interventions must move beyond generic digital literacy training to address age-specific barriers: developing elderly-centered interfaces with larger fonts and simplified navigation, establishing community-based peer learning programs where elderly teach each other, and integrating digital training with familiar activities like health monitoring or social services (Wang et al., 2019). However, enhancing digital literacy among hundreds of millions of elderly citizens represents a massive undertaking requiring sustained national investment in training programs, technical support, and device accessibility. Given the scale of resources and timeframe needed for such systemic transformation, digital communication will likely remain an inadequate substitute for in-person family support for the current elderly generation, particularly in rural and less developed regions where traditional support systems are most crucial (Hong et al., 2017; Fang et al., 2018).

### 4.3 Money support

The role of money support is multifaceted. In the 45–60 age group, depression tends to increase, which reflects a fundamental conflict between life stage expectations and dependency status. First, as elderly individuals in this age bracket often retain some work capacity, receiving money support from adult children may impact their self-esteem and hinder the development of self-fulfillment, potentially influencing their mental well-being (Li et al., 2022). The vulnerability model posits that low self-esteem can contribute to the onset of depression (Sowislo and Orth, 2013). Subsequent research has further substantiated that low self-esteem constitutes a risk factor for depression (Rehan et al., 2024). Individuals in this age category are typically not retired, and their earnings remain relatively high across the entire lifespan due to extensive work experience. According to Maslow’s hierarchy of needs theory, individuals in this age group are more inclined to prioritize the attainment of self-esteem and self-fulfillment over physiological support, such as financial assistance. For instance, working professionals in this age group who depend on financial support from adult children may experience what social individuals described as ‘losing face’ among peers who maintain financial independence.

From a social capital perspective (Putnam, 2000), this ‘loss of face’ extends beyond personal embarrassment to erode their bridging social capital—the professional networks and social connections that typically provide information, opportunities, and status. The transition from being a resource provider to a resource recipient within these networks fundamentally alters their social position, compounding the psychological distress. Consequently, they are at a higher risk of experiencing depression when their self-esteem and sense of self-fulfillment are unmet. Second, the power and bargaining model indicated control over financial resources determines family hierarchy and decision-making authority (Pyke, 1999). Intergenerational support for the elderly depends on the ability of control over family wealth, so that strong money support means that parents are losing their roles as wealth allocators in the family. And the weakening of family financial status is one of the factors that make the elderly depressed (Shu et al., 2021). The similar conclusion has been reached in a study of American old.

Third, although there might be expectations by older parents that they will receive financial transfers from their adult children in reason of filial piety (Chu et al., 2023), receiving financial support violates age-normative role expectations for 45–60 group. The 45–60 age group typically experiences peak earning capacity due to accumulated work experience and has not yet reached retirement age (60 in China). Individuals in this age bracket represent China’s ‘sandwich generation’ (Riley, 2005), accepting financial support during one’s productive years creates role incongruence—a mismatch between expected (provider) and actual (recipient) roles that generates psychological distress (Burke and Stets, 2009). Notably, while Korean middle-aged adults similarly experience filial pressure, their earlier retirement age (often 50–55) and cultural acceptance of early career transitions create a shorter window of role conflict (Lee et al., 2014). In contrast, China’s later retirement age (60) extends the period during which financial dependence conflicts with societal expectations of peak productivity.

However, for individuals aged 60–80, depression can be alleviated. As 60 is the retirement age for most individuals in China, there is a notable decrease in income for this demographic. Even in the absence of formal employment, individuals over the age of sixty typically experience a decline in work capacity due to physical limitations. A reduction in income becomes inevitable. In cases where pensions or income prove insufficient to sustain their previous standard of living, elderly parents who have exited the labor force must depend on their adult children for financial support (Wu and Li, 2014). Importantly, this transition facilitates a fundamental shift in role identity. Unlike working-age adults who struggle with recipient status, retirees have completed a socially sanctioned role transition from ‘provider’ to ‘elder deserving of care’ (Wang and Shultz, 2010). This role transformation aligns with developmental tasks of later life, where accepting care becomes part of successful aging rather than a failure of self-sufficiency (Baltes and Baltes, 1990).

Since the era of Confucius, the cultural concept of filial piety has transformed into the established norm dictating how the younger generation is anticipated to honor and respect their elders (Pan et al., 2016). The government also sought to emphasize the essential role that families play in upholding social order and ethics by enacting and reinforcing the citizens’ duty to provide financial support for their elderly parents, as mandated by law (Leung, 2008). According to Maslow’s Hierarchy of Needs theory, individuals in this age bracket prioritize physiological needs, such as financial security. Therefore, receiving financial support from their adult children would reduce their vulnerability to depression. Given that Chinese society has historically upheld the tradition of children providing financial support to their parents.

This pattern of financial support protecting against depression in later life appears consistent across diverse cultural contexts. Studies in both collectivist and individualist societies have found similar protective effects: in Japan, financial support from children reduced depression among elderly parents by 28% (Tiedt, 2013); in Mexico, regular remittances from adult children were associated with improved mental health among elderly recipients (Wong and Gonzalez-Gonzalez, 2010); even in individualist societies like Germany, financial transfers that maintain elderly autonomy showed protective effects against depression (Brandt and Deindl, 2013). These cross-cultural findings suggest that when financial support aligns with life stage expectations and preserves dignity, it serves as a buffer against late-life depression regardless of specific cultural contexts.

These cross-cultural findings suggest that when financial support aligns with life stage expectations and preserves dignity, it serves as a buffer against late-life depression regardless of specific cultural contexts. This highlights the need for differentiated financial support strategies: for the 45–60 group, interventions should preserve autonomy through collaborative approaches such as joint investment planning or family business partnerships that maintain their decision-making authority (Chen and Silverstein, 2000). For the 60–80 group, support should be delivered within culturally appropriate frameworks that emphasize filial respect while ensuring adequacy for maintaining quality of life (Zhang and Goza, 2006).

### 4.4 Goods support

Depression among individuals aged 45–80 can be mitigated when children provide goods support exceeding 6,000 yuan. These items serve as vessels for the conveyance of emotions, carrying concealed well-wishes that demonstrate care for middle-aged and elderly individuals (Polman and Maglio, 2017). This gesture conveys the filial piety of children, offering profound spiritual solace to the elderly.

Beyond the traditional aspects of filial culture, these items are visible markers of family care (Charles et al., 2013) to all. Social interaction among middle-aged and elderly individuals is often quite restricted, primarily consisting of casual conversations with peers, distinct from family communication (Mbao et al., 2021). These higher-value items can be mentioned, observed, and appreciated by those in their social circles during informal conversations. This social visibility effect appears particularly salient in collectivist cultures where ‘face’ and social comparison drive well-being. In contrast, research in individualistic societies shows that private financial transfers often have stronger effects than visible gifts, as they prioritize individual autonomy over social recognition (Lowenstein and Daatland, 2006). When surrounded by appreciation and comparisons from their peers, middle-aged and elderly individuals are more likely to cultivate a sense of self-contentment and self-fulfillment (Pardo-Cebrian et al., 2021), consequently decreasing the risk of depression.

These findings suggest that material support strategies should be tailored to social context and life stage: gifts should be selected not only for practical utility but for their capacity to enhance social standing and demonstrate family success, with consideration for regional economic standards and peer group expectations (Wu et al., 2008; Yan, 2009).

### 4.5 Theoretical integration

The differential effects of intergenerational support across age groups observed in this study can be comprehensively understood through the biopsychosocial model (Engel, 1977), which elucidates why identical support types produce divergent mental health outcomes by integrating biological aging, psychological development, and social-cultural factors.

Biologically, different age groups face distinct vulnerabilities—from the still-preserved physical vigor and cognitive abilities of midlife to the progressively accelerating decline in later years (Baltes and Smith, 2003). Psychologically, developmental tasks shift from achievement-focused to acceptance-oriented across the lifespan (Diehl et al., 2015). Socially, cultural scripts define age-appropriate dependencies within the Chinese filial piety framework (Cheng and Chan, 2006), although these cultural scripts are increasingly contested by modernization forces.

The biopsychosocial model reveals that support effectiveness depends not on the support type per se, but on its alignment across all three levels. Financial support succeeds for the 60–80 group because it addresses biological vulnerabilities (health needs), aligns with psychological tasks (accepting care), and fulfills social expectations (filial piety). It fails for the 45–60 group by threatening biological stress systems (status loss), violating psychological needs (self-actualization), and breaching social norms (provider role). This integrative framework advances beyond examining isolated mechanisms to reveal how intergenerational support operates as a complex system where biological aging, psychological development, and cultural contexts dynamically interact to shape mental health outcomes (Antonucci et al., 2014). This multi-level perspective explains why interventions must be age-tailored: enhancing digital literacy might help the 45–60 group maintain productive engagement, while ensuring dignified financial support better serves the 60–80 group, and facilitating co-residence arrangements benefits the 80 + cohort.

### 4.6 Methodological considerations

Methodological heterogeneity across studies may partially account for divergent findings regarding intergenerational support effects. Depression measurement varies considerably—while we used the CES-D-10, some other studies often employ the Geriatric Depression Scale (GDS) or PHQ-9, which may capture different aspects of depressive symptomatology (Bjørkløf et al., 2013).

More critically, operationalization of support differs substantially: our study examined frequency and monetary thresholds, while others measure perceived quality, satisfaction, or reciprocity of support (Li and Yang, 2021). For instance, studies reporting positive effects of financial support often assess subjective adequacy rather than absolute amounts, potentially explaining why the same support type yields opposing outcomes.

Additionally, temporal frameworks vary—cross-sectional designs like ours capture associations at one time point, while longitudinal studies revealing positive effects may reflect adaptation processes over time (Schwartz et al., 2019). These methodological variations underscore the need for standardized measurement approaches in cross-cultural intergenerational support research.”

### 4.7 Limitations and future directions

There are some limitations that need to be considered. First, potential endogeneity issues arise from both reverse causality and omitted variables. The cross-sectional design cannot capture the bidirectional relationship between depression and intergenerational support. Additionally, unmeasured factors such as parent–child relationship quality, personality traits, or health shocks may simultaneously influence both support patterns and mental health outcomes. Moreover, our pre-pandemic data (CHARLS, 2018) cannot capture how major societal disruptions like pandemic may have altered elderly attitudes toward online communication and intergenerational support patterns. Future panel studies tracking both support patterns and mental health trajectories over time are essential to establish temporal precedence and clarify these reciprocal dynamics.

Second, our reliance on self-reported measures introduces potential biases. Depression symptoms may be underreported due to stigma in Chinese culture, while support received might be over- or underestimated based on respondents’ emotional states or social desirability concerns (Huang et al., 2019). Future mixed-methods research combining quantitative surveys with qualitative interviews could illuminate how elderly individuals subjectively experience and interpret different support types, revealing nuances that standardized measures miss.

Third, our analysis, while revealing age-specific patterns, could not fully explore the complex heterogeneity in intergenerational support effects: urban–rural disparities that may fundamentally alter support dynamics due to differences in pension coverage, healthcare access, and migration patterns; three-way interactions between support type, age, and gender that may reveal how traditional gender roles shape support effectiveness across the life course. Such multidimensional analyses would provide more nuanced understanding of which elderly subgroups benefit most from specific support types, enabling targeted policy interventions rather than one-size-fits-all approaches.

## 5 Conclusion

Offline companionship is highly effective in alleviating depression among older adults. The current generation of middle-aged and elderly individuals has not fully embraced online companionship with their children. Second, money support must be provided with careful consideration of the elderly’s employment and financial situation due to its multifaceted effects. The provision of high-value goods significantly reduces depression in middle-aged and elderly individuals. In summary, online companionship did not demonstrate a substantial positive effect in alleviating depression. For individuals aged 45–60, children can offer valuable items to help mitigate parental depression. For those aged 60–80, both financial support and the provision of valuable goods are essential. Seniors aged 80 and above require additional offline companionship.

## Data Availability

Publicly available datasets were analyzed in this study. This data can be found at: https://charls.pku.edu.cn/.
