# Supplementary material for: Age-specific associations between intergenerational support from children and depression in middle-aged and elderly Chinese: results from the China health and retirement longitudinal study
Source: Front Psychol. 2025 Jul 17;16:1621444. doi: 10.3389/fpsyg.2025.1621444 (PMC12311948; doi:10.3389/fpsyg.2025.1621444)
Supplement: Supplementary file 1 [file Data_Sheet_1.PDF]

supplementary file1

CSED Scale Score Sheet

[INTRO: The 10 items below refer to how you have felt and behaved during the last week. Every item has the same selective answers including rarely or none of the time, some, occasionally. And most or all of the time. Choose the appropriate response]

Item	Score
**①I was bothered by things that don't usually bother me.**<br>1.Rarely or none of the time<br>2.Some or a little of the time<br>3.Occasionally or a moderate amount of the time<br>4.Most or all of the time	“1”=0 score<br>“2”=1 score<br>“3”=2 score<br>“4”=3 score
**②I had trouble keeping my mind on what I was doing.**<br>1.Rarely or none of the time<br>2.Some or a little of the time<br>3.Occasionally or a moderate amount of the time<br>4.Most or all of the time	“1”=0 score<br>“2”=1 score<br>“3”=2 score<br>“4”=3 score
**③I felt depressed.**<br>1.Rarely or none of the time<br>2.Some or a little of the time<br>3.Occasionally or a moderate amount of the time<br>4.Most or all of the time	“1”=0 score<br>“2”=1 score<br>“3”=2 score<br>“4”=3 score
**④I felt everything I did was an effort.**<br>1.Rarely or none of the time<br>2.Some or a little of the time<br>3.Occasionally or a moderate amount of the time<br>4.Most or all of the time	“1”=0 score<br>“2”=1 score<br>“3”=2 score<br>“4”=3 score
**⑤I felt hopeful about the future.**<br>1.Rarely or none of the time<br>2.Some or a little of the time<br>3.Occasionally or a moderate amount of the time<br>4.Most or all of the time	“1”=3 score<br>“2”=2 score<br>“3”=1 score<br>“4”=0 score
**⑥I felt fearful.**<br>1.Rarely or none of the time<br>2.Some or a little of the time<br>3.Occasionally or a moderate amount of the time<br>4.Most or all of the time	“1”=0 score<br>“2”=1 score<br>“3”=2 score<br>“4”=3 score
**⑦My sleep was restless.**<br>1.Rarely or none of the time<br>2.Some or a little of the time<br>3.Occasionally or a moderate amount of the time<br>4.Most or all of the time	“1”=0 score<br>“2”=1 score<br>“3”=2 score<br>“4”=3 score
**⑧I was happy.**	“1”=3 score

1.Rarely or none of the time<br>2.Some or a little of the time<br>3.Occasionally or a moderate amount of the time<br>4.Most or all of the time	“2”=2 score<br>“3”=1 score<br>“4”=0 score
**⑨I felt lonely.**<br>1.Rarely or none of the time<br>2.Some or a little of the time<br>3.Occasionally or a moderate amount of the time<br>4.Most or all of the time	“1”=0 score<br>“2”=1 score<br>“3”=2 score<br>“4”=3 score
**⑩I could not get ”going”.**<br>1.Rarely or none of the time<br>2.Some or a little of the time<br>3.Occasionally or a moderate amount of the time<br>4.Most or all of the time	“1”=0 score<br>“2”=1 score<br>“3”=2 score<br>“4”=3 score
**CSED Scale Score**	sum of the individual items

supplementary file2

Control covariates	measurement
gender	1 = male, 2 = female
place of residence	1= the center of city/town, 2=combination zone between urban and rural areas, 3=village, 4= special area
education level	1= illiterate, 2= primary School, 3= junior high school, 4= high school or specialist, 5= bachelor's degree or above
marital status	1= married and cohabiting, 2= married but not currently cohabiting for specific reasons, 3= divorced or widowed, 4= never married
Social activity	whether participants engaged in any social activities in the past month (1=Yes, 2=No), such as socializing with friends, participating in club activities, attending training courses, or using the Internet, among others.
pensions	whether participants were currently receiving or expected to receive pensions in the future (1=Yes, 2=No)
health status	asking respondents to rate their health status on a 5-point Likert scale: 1 = very good, 2 = good, 3 = fair, 4 = poor, and 5 = very poor.

supplementary file3

The robustness test of Table 2

Variables	Coef.	Std. Err.	z	P>z	[95% Conf.	Interval]
offline companionship	-0.025	0.007	-3.460	0.001	-0.039	-0.011
online companionship	0.046	0.017	2.780	0.005	0.013	0.078
money support	0.008	0.020	0.390	0.699	-0.031	0.046
goods support	-0.049	0.026	-1.910	0.056	-0.099	0.001
sex	0.304	0.027	11.190	0.000	0.251	0.357
age	-0.178	0.025	-7.180	0.000	-0.227	-0.130
residence	0.058	0.019	3.030	0.002	0.020	0.096
education	-0.081	0.017	-4.850	0.000	-0.114	-0.049
marital status	-0.016	0.015	-1.040	0.299	-0.046	0.014
health status	0.422	0.014	30.730	0.000	0.395	0.449
social activity	0.004	0.002	1.930	0.054	0.000	0.009
pension insurance	0.184	0.041	4.450	0.000	0.103	0.265
Observation				11398		
Pseudo R2				0.1071		

The robustness test of model 1 in Table 3

Variables	Coef.	Std. Err.	z	P>z	[95% Conf.	Interval]
offline companionship	-0.013	0.012	-1.120	0.261	-0.037	0.010
online companionship	0.062	0.024	2.570	0.010	0.015	0.110
money support	0.050	0.030	1.640	0.101	-0.010	0.110
goods support	-0.042	0.039	-1.070	0.286	-0.119	0.035
sex	0.327	0.041	8.030	0.000	0.247	0.407
residence	0.053	0.028	1.860	0.062	-0.003	0.108
education	-0.133	0.025	-5.270	0.000	-0.182	-0.083
marital status	0.091	0.029	3.180	0.001	0.035	0.147
health status	0.417	0.021	20.180	0.000	0.377	0.458
social activity	0.004	0.003	1.110	0.267	-0.003	0.010
pension insurance	0.077	0.062	1.240	0.214	-0.045	0.200
Observation				4893		
Pseudo R2				0.1200		

The robustness test of model 2 in Table 3

Variables	Coef.	Std. Err.	z	P>z	[95% Conf.	Interval]
offline companionship	-0.015	0.010	-1.500	0.134	-0.034	0.005
online companionship	0.040	0.024	1.640	0.101	-0.008	0.087
money support	-0.051	0.028	-1.850	0.065	-0.105	0.003
goods support	-0.050	0.036	-1.390	0.164	-0.121	0.021
sex	0.340	0.039	8.750	0.000	0.264	0.416
residence	0.086	0.028	3.050	0.002	0.031	0.140
education	-0.043	0.024	-1.820	0.069	-0.090	0.003
marital status	-0.035	0.020	-1.770	0.077	-0.073	0.004
health status	0.422	0.019	21.910	0.000	0.384	0.460
social activity	0.004	0.003	1.150	0.249	-0.002	0.009
pension insurance	0.287	0.059	4.860	0.000	0.172	0.403
Observation				5780		
Pseudo R2				0.1077		

The robustness test of model 3 in Table 3

**Variables**	**Coef.**	**Std. Err.**	**z**	**P>z**	**[95% Conf.**	**Interval]**
offline companionship	-0.084	0.029	-2.850	0.004	-0.142	-0.026
online companionship	-0.076	0.082	-0.930	0.353	-0.236	0.084
money support	0.044	0.085	0.520	0.606	-0.122	0.210
goods support	-0.154	0.106	-1.450	0.147	-0.362	0.054
sex	0.083	0.127	0.650	0.515	-0.167	0.333
residence	-0.179	0.083	-2.160	0.031	-0.342	-0.016
education	0.055	0.076	0.730	0.465	-0.093	0.204
marital status	-0.146	0.065	-2.230	0.026	-0.273	-0.018
health status	0.372	0.067	5.560	0.000	0.241	0.503
social activity	-0.003	0.009	-0.290	0.774	-0.021	0.016
pension insurance	0.279	0.187	1.500	0.135	-0.087	0.645
Observation				725		
Pseudo R2				0.0705		

The robustness test of model 1 in Table 4

**Variables**	**Coef.**	**Std. Err.**	**z**	**P>z**	**[95% Conf.**	**Interval]**
offline companionship	-0.013	0.012	-1.100	0.272	-0.037	0.010
online companionship	-0.058	0.172	-0.340	0.734	-0.395	0.278
money support	-0.100	0.329	-0.300	0.761	-0.746	0.546
goods support	-0.270	0.279	-0.970	0.334	-0.817	0.278
money support*goods support	0.139	0.178	0.780	0.436	-0.211	0.488
online companionship*money support	0.076	0.062	1.220	0.221	-0.046	0.199
online companionship* goods support	0.105	0.108	0.970	0.332	-0.107	0.317
online companionship*money support* goods support	-0.064	0.067	-0.950	0.341	-0.195	0.068
sex	0.328	0.041	8.050	0.000	0.248	0.408
residence	0.054	0.028	1.900	0.057	-0.002	0.109
education	-0.132	0.025	-5.240	0.000	-0.181	-0.083
marital status	0.091	0.029	3.180	0.001	0.035	0.147
health status	0.417	0.021	20.160	0.000	0.376	0.457
social activity	0.004	0.003	1.100	0.272	-0.003	0.010
pension insurance	0.076	0.062	1.220	0.221	-0.046	0.199
Observation				4893		
Pseudo R2				0.1202		

The robustness test of model 2 in Table 4

Variables	Coef.	Std. Err.	z	P>z	[95% Conf.	Interval]
offline companionship	-0.014	0.010	-1.450	0.147	-0.034	0.005
online companionship	0.037	0.024	1.510	0.132	-0.011	0.084
money support	0.031	0.089	0.350	0.728	-0.143	0.205
goods support	0.027	0.087	0.310	0.758	-0.144	0.198
money support*goods support	-0.045	0.046	-0.970	0.331	-0.135	0.045
sex	0.339	0.039	8.720	0.000	0.263	0.415
residence	0.085	0.028	3.030	0.002	0.030	0.140
education	-0.043	0.024	-1.810	0.071	-0.090	0.004
marital status	-0.035	0.020	-1.760	0.079	-0.073	0.004
health status	0.422	0.019	21.920	0.000	0.385	0.460
social activity	0.003	0.003	1.150	0.250	-0.002	0.009
pension insurance	0.284	0.059	4.790	0.000	0.168	0.400
Observation			5780			
Pseudo R2			0.1078			
